# NMR Study on the Inclusion Complexes of β-Cyclodextrin with Isoflavones

**DOI:** 10.3390/molecules21040372

**Published:** 2016-03-28

**Authors:** Rui Zhao, Corine Sandström, Haiyang Zhang, Tianwei Tan

**Affiliations:** 1 School of Food and Chemical Engineering, Beijing Engineering and Technology Research Center of Food Additives, Beijing Higher Institution Engineering Research Center of food additives and Ingredients, Beijing Key Laboratory of Flavor Chemistry, Beijing Technology and Business University, Beijing 100048, China; 2 Department of Chemistry and Biotechnology, Uppsala BioCenter, Swedish University of Agricultural Sciences, P. O. Box 7015, Uppsala SE-750 07, Sweden; corine.sandstrom@slu.se; 3 College of Life Science and Technology, Beijing University of Chemical Technology, Beijing 100029, China; zhanghy@ustb.edu.cn (H.Z.); twtan@mail.buct.edu.cn (T.T.)

**Keywords:** β-cyclodextrin, flavonoids, NMR, interaction

## Abstract

The structure of the inclusion complexes of β-cyclodextrin (β-CD) with daidzein and daidzin in D_2_O were investigated using NMR spectroscopy. For the β-CD and daidzein system, two types of 1:1 complexes were formed with the daidzein deeply inserted into the CD cavity with different orientations. For the β-CD/daidzin system, a 1:1 complex was formed with the flavonoid part of daidzin entering the CD cavity from the wide rim. The inclusion complexes determined by NMR were constructed using molecular docking. Furthermore, the mixture of puerarin, daidzein and daidzin, which are the major isoflavonoid components present in *Radix puerariae*, was analyzed by diffusion-ordered spectroscopy (DOSY) alone and upon addition of β-CD in order to mimic chromatographic conditions and compare their binding affinities.

## 1. Introduction

**Figure 1:**
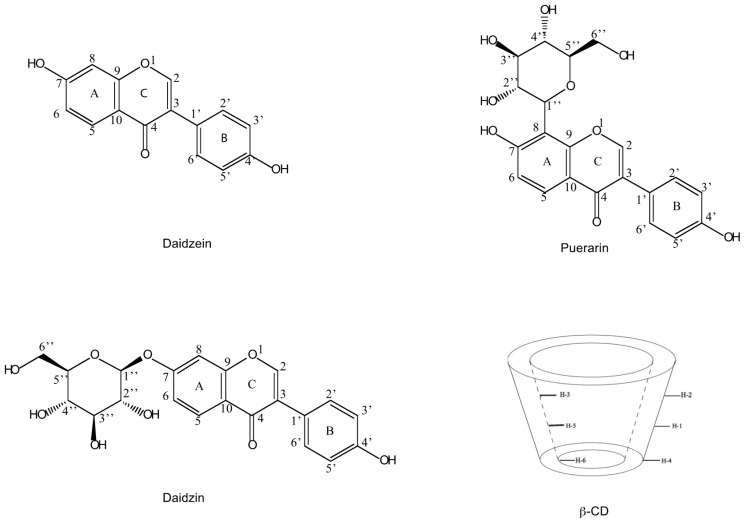
Structures of daidzein (**1**), daidzin (**2**) and puerarin (**3**) and a schematic representation of β-CD.

Isoflavones from *Radix puerariae* have been widely used as antipyretic, antidiarrhoetic, diaphoretic, and antiemetic agents in traditional Chinese medicine (TCM) [1,2,3,4]. Puerarin (daidzein 8-C-glucoside), daidzin (daidzein 7-*O*-glucoside) and daidzein (Figure 1) are the major isoflavonoids present in *Radix puerariae* [5]. In recent years, there has been an increasing interest in studying the individual active herb components instead of using the whole herb or the whole herb extract as the chemotherapeutic agent. Although the traditional separation methods such as extraction and precipitation can be applied with low cost, these approaches are restricted because of low recoveries and purities. Thus, there is a strong need of developing highly sensitive and selective methods for the isolation and characterization of these compounds.

β-Cyclodextrin (β-CD), coupled for example to agarose or silica gel media, is widely used in TCM purification. Having a shape like truncated cone with a hydrophilic external surface and a hydrophobic central cavity, CDs are capable of creating inclusion complexes with various molecules by including them into the cavity [6,7,8,9,10]. The driving forces for complex formation are van der Waals, hydrophobic or hydrogen bond interactions [11,12].

Thus, chromatography with β-CD as the ligand could provide a way of purifying the mixture of isoflavones [13,14,15]. We had recently studied the formation of inclusion complex between puerarin and β-CD using proton NMR chemical shifts and rotating frame nuclear Overhauser effect spectroscopy (ROESY) experiments [16] and the existence of hydrogen bonding interaction was proposed from a molecular dynamic study [17,18] and later on demonstrated using NMR data of hydroxy protons [19]. To our knowledge however, the interactions of β-CD with the two other major components of isoflavones, daidzein and daidzin, or the potential use of β-CD to separate the three components as a mixture has not yet been investigated experimentally.

In the present work, the structures of the inclusion complexes of β-CD with daidzein and daidzin (Figure 1), were studied in D_2_O using 1D and 2D NMR experiments. These structures were then optimized using molecular docking. Moreover, the potential of diffusion-ordered spectroscopy (DOSY) [20,21,22], sometimes also described as “in tube NMR chromatography”, to separate a mixture of daidzein, daidzin and puerarin based on their binding affinity to β-CD was also investigated.

## 2. Results and Discussion

Due to the poor solubility of daidzein (**1**) and daidzin (**2**) in water and the absence of complex formation in organic solvents, the NMR experiments were carried out at pH 12.0 in D_2_O. ^1^H-NMR spectra for the daidzein/β-CD and daidzin/β-CD complexes were recorded in the temperature ranges of 293–333 K. An increase in temperature resulted in broadening of signals and in spectral overlap, but also in degradation of the guest molecules. The optimum temperature was thus found to be 30 °C.

### 2.1. Stoichiometry of the Complexes

**Figure 2:**
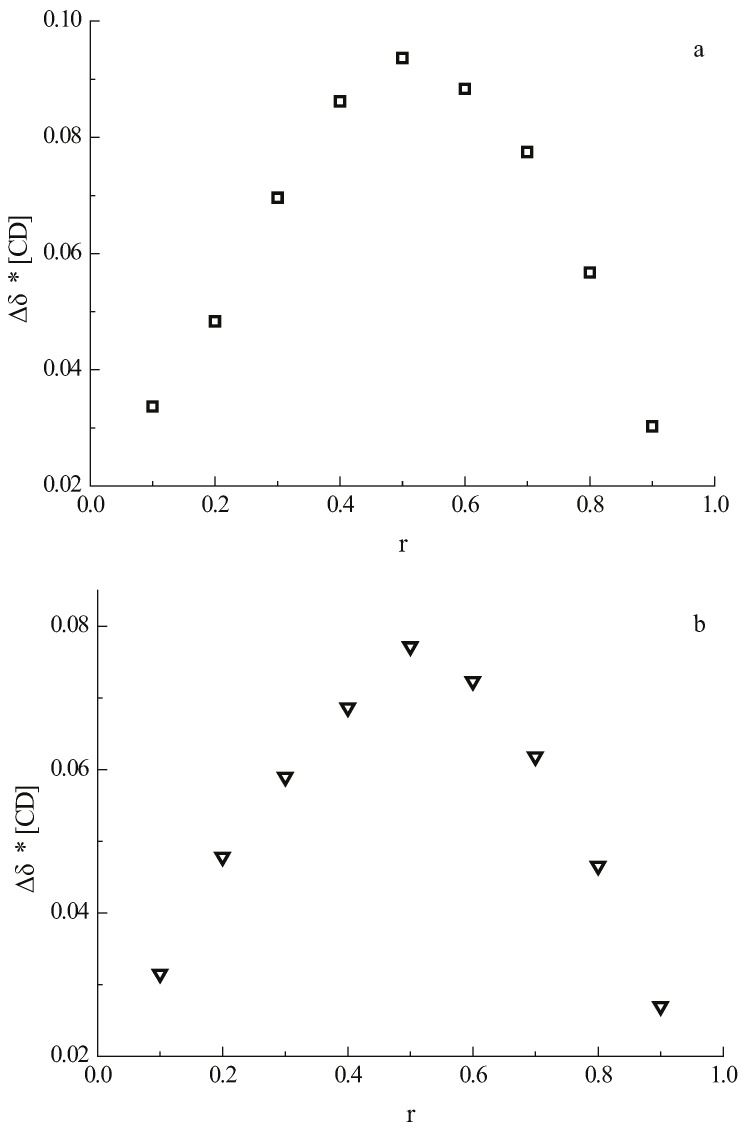
Job’s plot using the chemical shift of the H-5 signal of β-CD of (**a**) the complex of daidzein with β-CD; (**b**) the complex of daidzin with β-CD. “r” is the molar fraction of β-CD in the guest/β-CD mixture.

The stoichiometries of the complexes of **1** and **2** with β-CD were determined using the continuous variation method (Job’s method) [23,24]. The guest and the host molecules were distributed among nine NMR tubes, varying the molar ratio from 1:9 to 9:1 at a constant total concentration of 2 mM. The complexation-induced shifts of H-5 of β-CD are shown on the Job plot in Figure 2 where the apex at 0.5 indicates the formation of a 1:1 complex between β-CD and daidzein as well as between β-CD and daidzin.

### 2.2. Structure of the Inclusion Complexes of Daidzein and Daidzin with β-CD

#### 2.2.1. Proton Chemical Shifts

The proton signals of daidzein and daidzin were assigned in D_2_O at 30 °C on the basis of ^1^H-^1^H COSY spectra and the assignment of β-CD was according to the literature [25]. The inclusion of daidzein and daidzin into the β-CD cavity is evidenced by the chemical shift changes observed in the ^1^H-NMR spectra of both β-CD and the guest molecules. Only one set of resonances was observed indicating that the complexation occurs under fast exchange conditions relative to the NMR time scale.

Upon addition of daidzein to β-CD, the H-3 and H-5 signals of β-CD positioned on the inner surface of β-CD, as well as the H-6 signal located on the cavity rim at the narrow end of the molecule, showed significant upfield shift (Table 1). The largest chemical shift changes were observed for H-5 (−0.11 ppm) followed by H-6 (−0.09 ppm) and H-3 (−0.06 ppm). These upfield shifts are due to the ring current effect of the aromatic systems of daidzein included into the CD cavity [26,27]. The chemical shifts of the proton signals of daidzein were also affected by the presence of β-CDs (Table 1). Thus, H-2 in the C ring, H-8 in the A ring and H-2′,6′ in the B ring were shielded while H-5 and H-6 in the A ring were deshielded, indicating that the C ring and parts of A and B rings of daidzein entered into the cavity of β-CD.

For the daidzin/β-CD system, the chemical shifts of the protons of β-CD and daidzin alone as well as the complexation induced shifts are listed in Table 2. The shielding of H-5, H-3 and H-6 in β-CD and H-2 and H-2′,6′ in daidzin indicated that daidzin is entering the CD cavity. However, the complexation induced shifts for the β-CD/daidzin complex were smaller than for the β-CD/daidzein complex suggesting a weaker interaction between β-CD and daidzin if compared to β-CD and daidzein.

#### 2.2.2. ROESY Experiments

**Figure 3:**
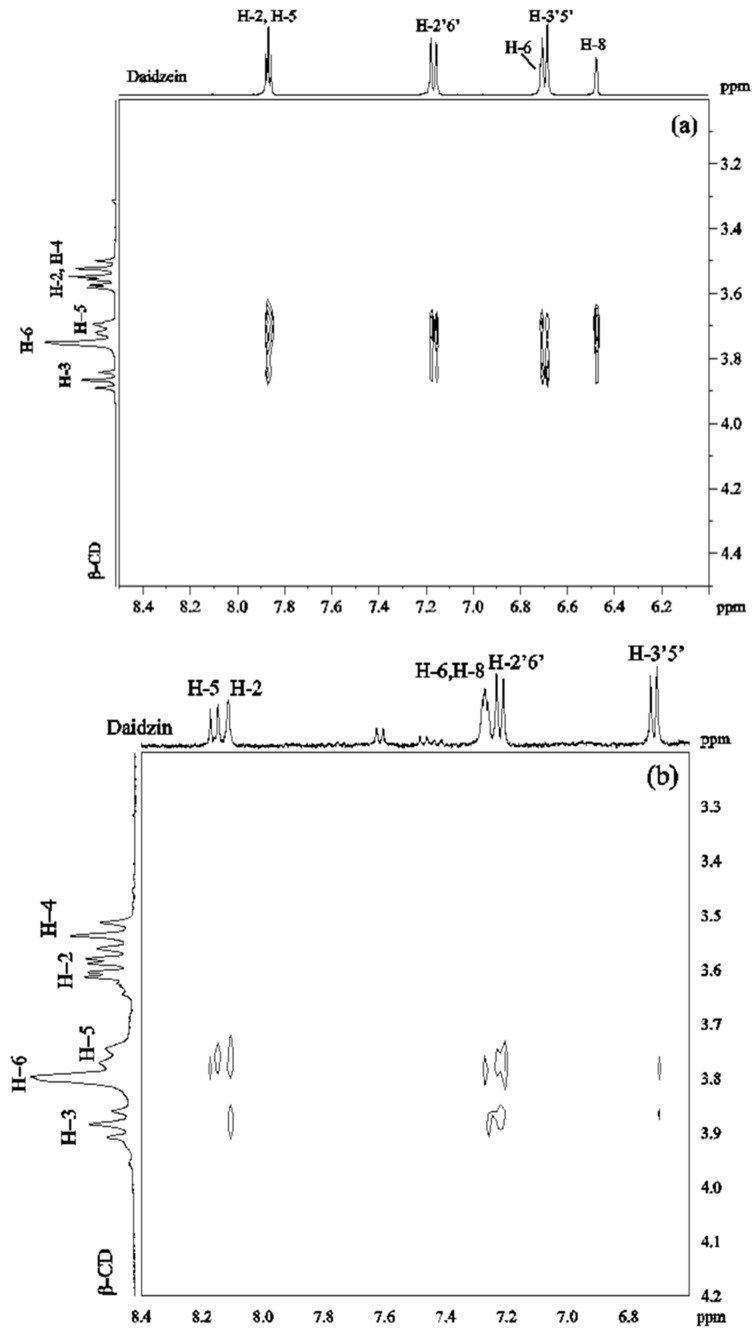
2D ROESY spectrum of (**a**) daidzein/β-CD (1:1) in D_2_O at 30 °C; (**b**) daidzin/β-CD (1:1) in D_2_O at 30 °C.

ROESY spectra were obtained in D_2_O solutions containing equimolar amounts of β-CD/daidzein (2 mM) and β-CD/daidzin (2 mM). The 2D ROESY spectrum of the complex of daidzein and β-CD (Figure 3a) showed strong ROEs between H-2, H-5 of daidzein and H-5 of β-CD. Medium ROEs were observed between H-2, H-5 of daidzein and H-3 of β-CD, between H-2′6′ of daidzein and H-5 of β-CD, between H-3′5′ or/and H-6 of daidzein and H-5, H-3 of β-CD, as well as between H-8 of daidzein and H-5 of β-CD. There were weak ROEs between H-8 of daidzein and H-3 of β-CD and between H-2′6′ of daidzein and H-3 of β-CD. No ROE was observed between daidzein and H-4 of β-CD. These ROEs suggest that two different types of complexes are formed which differ in the orientation of daidzein inside the cavity of β-CD. In one type of complex, the B ring of daidzein is approaching to the wide rim of β-CD, whereas in the other type the A ring is closed to the wide rim.

In the ROESY spectrum of a 1:1 solution of daidzin and β-CD (Figure 3b), medium ROE correlations were found between H-2, H-2′6′ of daidzin and H-5 of β-CD. Weak ROEs were found between H-5, H-3′5′ of daidzin and H-5 of β-CD, between H-2, H-8 and H-2′6′ of daidzin and H-3 of β-CD and between H-3′5′ of daidzin and H-5 of β-CD. There was no cross-peak between H-3′5′ of daidzin and H-3 of β-CD. A weak ROE was observed between β-CD and either H8 or H6 of daidzin which could not be distinguished due to signal overlapping. These ROEs showed that ring C and parts of rings A and B of daidzin are included in the cavity of β-CD with ring B on the narrow side of the cavity.

Thus, the NMR data show that the aromatic rings in daidzein (**1**), daidzin (**2**) and puerarin (**3**) (previously investigated in ref. [19]), are forming inclusion complexes with β-CD. Due to its smaller molecular size without the hydrophilic glucose residue, daidzein not only formed a stronger inclusion complex with β-CD if compared to the other two compounds, but it also can form two different types of inclusion complexes.

### 2.3. DOSY of Complexes of Isoflavones **1**–**3** with β-CD

**Figure 4:**
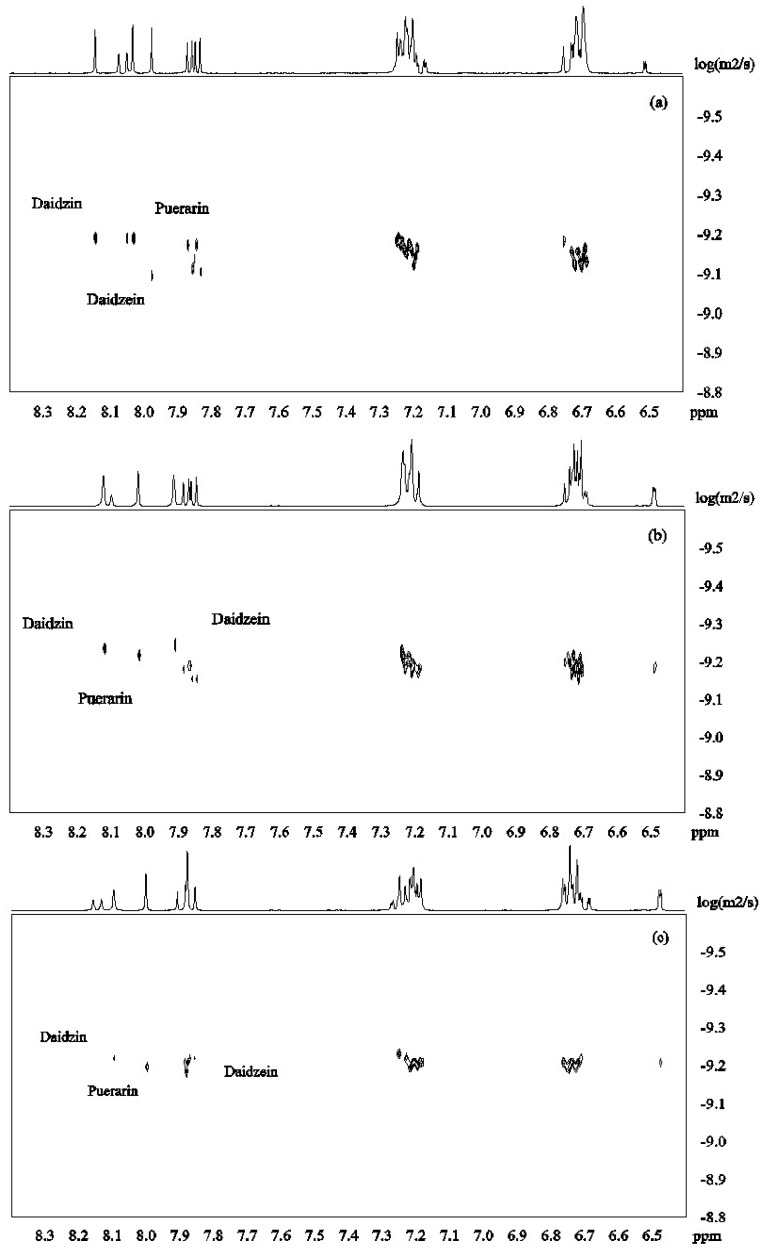

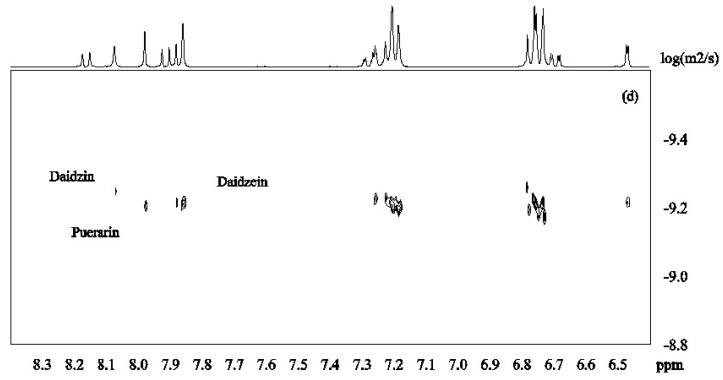
DOSY spectra of daidzein, daidzin and puerarin ((**a**): 2.0 mM for each); in the presence of β-CD ((**b**): β-CD 2.0 mM; (**c**): β-CD 4.0mM; (**d**): β-CD 6.0 mM in D_2_O, 30 °C).

DOSY spectra for the mixture of isoflavones **1**–**3** alone and in the presence of β-CD were recorded at pH 12 in D_2_O (Figure 3). Figure 4a shows that the signals from daidzein were separated from those from the two other compounds due to faster diffusion caused by the smaller molecular mass. Puerarin and daidzin which have same molecular weight had similar apparent diffusion coefficients (Figure 4a). Diffusion coefficients are highly responsive to shape factors but even though puerarin is potentially more spherical than daidzin, similar diffusion rate were observed.

β-CD was then added to the mixture of isoflavones **1**–**3** in order to investigate its potential to separate the three compounds in the DOSY experiments based on the strength of the inclusion complexes. Upon addition of β-CD (2mM) to the solution of isoflavones **1**–**3** (2 mM for each compound), daidzein showed the slowest diffusion because of its more favored inclusion complex formation with β-CD (Figure 4b). This is in good agreement with the results obtained from ROESY and H^1^ NMR experiments. When the concentration of β-CD was increased to 4 mM, the apparent diffusion coefficients of both daidzin and puerarin decreased with daidzin showing a slightly lower diffusion rate than puerarin (Figure 4c), indicating that daidzin was binding slightly more strongly to β-CD than puerarin. The structural differences between daidzin (**2**) and puerarin (**3**) are the additional hydroxyl group in puerarin and the position of attachment of the sugar on the ring. While an additional hydroxyl group is not favorable to the formation of inclusion complex governed by hydrophobic interaction, the larger steric effect of the sugar unit when attached to puerarin (**3**) if compared to daidzin (**2**) is probably the main factor responsible for the slightly lower binding affinity of puerarin [28].

The observed diffusion coefficients *D_obs_* of **1**–**3** with and without addition of β-CD and the association constants *K* were estimated from the DOSY experiment. Since the systems investigated in this study are under fast equilibrium between free and bound states on the NMR time scale, *D_obs_* is the weighted average of those of the free and bound molecules (Equation (1)). *K* can be calculated using single-point procedure [29,30] (Equation (2) [31]) on the premise of known mole fraction χ of the bound guest χ_b_. In this procedure, it is assumed that the diffusion coefficient of the host-guest complex is the same as that of the host molecule:

D_obs_ = χ D_bound_ + (1 − χ) D_free _ (1)

K = χ_b_/(1 − χ_b_) ([H]_0_*−* χ_b_ [G]_0_) (2)

In Equation (1), D_obs_ is the observed diffusion coefficient while D_free_ and D_bound_ are the diffusion coefficients of free and bound guest molecules, respectively. In Equation (2), [H]_0_ and [G]_0_ are the total concentration of the host and guest respectively.

The diffusion coefficients (D) measured at pH 12 in D_2_O and the association constants are reported in Table 3. Although these values, obtained by the single-point approximation method, result in large uncertainty, they can still be used to compare the relative binding affinity of the isoflavones **1**–**3** with β-CD. In presence of β-CD, the three compounds showed a decrease in diffusion rates confirming the formation of inclusion complexes deduced from proton chemical shifts and ROESY experiments. Among these three compounds, the binding strength of daidzein is highest followed by daidzin and by puerarin. The binding constant of puerarin was lower than the one reported in [19] because different pH solutions were used. While the isoflavones and the β-CD hydroxyl groups will be partially deprotonated at pH 12, it will most probably not influence the structure of the inclusion complexes which are mainly stabilized by hydrophobic interaction. We have shown previously using NMR of hydroxy protons that the inclusion complex between puerarin and β-CD was not stabilized by strong hydrogen bond interactions [19]. It has also been shown that trifluoperazine for example can form complexes with β-CD under unionized forms. In this case, the binding interaction was mainly due to van der Waals forces and hydrophobic interactions [32]. It has however also been shown that most inclusion complexes have best stability at pH 7 but due to the low solubility of daidzein, we were not able to perform the NMR studies at this pH.

### 2.4. Docking of the Complexes of Isoflavones with β-CD

**Figure 5:**
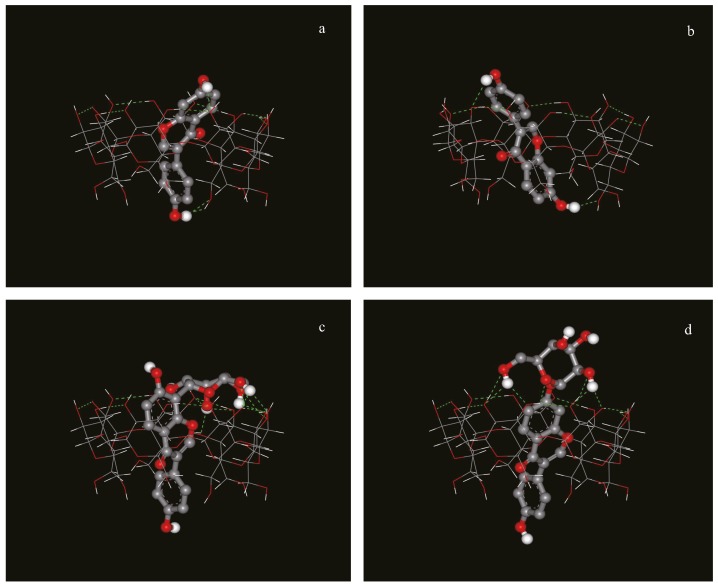
Possible structure of the inclusion complex between (**a**): daidzein and β-CD, type I; (**b**): daidzein and β-CD, type II; (**c**): puerarin and β-CD; (**d**): daidzin and β-CD obtained with the program Autodock 4.0).

In order to confirm the experimental data obtained by NMR, molecular modeling of the complexes was carried out. These studies revealed that a preferred final relative orientation for all the complexes investigated occurs in spite of the different initial configurations arbitrarily imposed. After a cluster analysis of the docking results, the three complexes with the lowest docked energies were chosen, and shown in Figure 5.

Daidzein penetrated into the cavity of β-CD, and there were two types of complexes with different orientations of daidzein in the cavity, consistent with the NMR data. For daidzin and puerarin, the A and C rings were inserted into the cavity of β-CD from the wide rim in a similar manner, while the B rings of both compounds extended from the narrow rim and the glucose unit protruded outside of the β-CD’s cavity. Thus, the structure of the inclusion complex for dadzin and puerarin after the docking calculations are also in very good agreement with the structures that were deduced from the NMR data.

## 3. Experimental Section

### 3.1. Materials

Puerarin, daidzein and daidzin were purchased from the National Institute for the Control of Pharmaceutical and Biological Products, Beijing, China and β-cyclodextrin was purchased from Sigma–Aldrich Inc., Beijing, China.

### 3.2. Sample Preparation

β-CD was freeze-dried before used while the guest molecules, while puerarin, daidzein and daidzin, were used as supplied. All compounds were dissolved in a solution of Na_2_HPO_4_/NaOH buffer (pH 12). The samples of β-CD complexes were prepared to have 2.0 mM concentrations and a 1:1 molar ratio for both host and guest molecules. NMR spectra of β-CDs and of puerarin, daidzein and daidzin alone were recorded at the same concentrations as those used to study the complexes.

### 3.3. NMR

All NMR experiments were performed on a DRX 400 MHz spectrometer (Bruker, Karlsruhe, Germany) using a 5 mm ^1^H/^13^C/^15^N/^31^P QNP probe equipped with z-gradient. For experiments in D_2_O, the chemical shifts were referenced relative to the residual DOH signal set at δ_H_ 4.70 ppm at X C. Two-dimensional correlation spectroscopy (COSY), Total correlation spectroscopy (TOCSY) and ROESY NMR spectra were acquired using standard pulse sequences from the Bruker library. A spectral width of 3205 Hz with 4K data points in t2 and 512 in t1 were used. The relaxation delay between successive pulse cycles was 1.5 s for COSY and TOCSY and 2 s for ROESY. Mixing times of 80 ms and 200 ms were used for TOCSY and ROESY, respectively.

For DOSY experiments, data acquisition and analysis were performed using the Bruker TOPSPIN software (version 1.3, Bruker). The DOSY experiments were performed using the ledbpgp2s pulse sequence from the Bruker library, with stimulated echo, longitudinal eddy current compensation, bipolar gradient pulses, and two spoil gradients using 16 different gradient values varying from 2 to 95% of the maximum gradient strength. 100 ms diffusion time was chosen for samples in D_2_O. The gradient length was set to 2.2 ms for this system. Processing was achieved using 2048 points in the F2 dimension and 512 points in F1. An exponential window function with 1 Hz line broadening was applied in the F2 dimension prior to Fourier transformation. After baseline correction, the diffusion dimension was processed with the DOSY processing program (Bruker TopSpin software 2.0). A logarithmic scaling was applied in the diffusion axis, and a noise sensitivity factor of 4 and line width factor of 2 were used. The fitting of the diffusion dimension in the 2D-DOSY spectra was obtained using a single exponential fit (Nexp = 1). The DOSY experiment was carried out twice and for two different sample preparations.

### 3.4. Molecular Modeling

AutoDock 4.0 [33] was used to investigate the binding mode of a guest to its host. The structure of host (β-CD) was extracted from the Protein Data Bank (PDB code: 2V8L). Coordinates of guest molecules (puerarin, daidzin, and daidzein) were generated and energy-minimized using the Dundee PRODRG Server [34]. Partial charges of host and guests were assigned using the Gasteiger method with the aid of AutoDockTools. The grid spacing was 0.0375 nm in each dimension, and each grid map consisted of a 40 × 40 × 40 grid point. The Lamarckian genetic algorithm (LGA) was adopted to model the interaction between host and guest and to search for a globally optimized conformation. During each docking experiment ten runs were carried out. The other parameters were set as default values. After a cluster analysis to docking results, the complex with the lowest docked energy was selected to be the representative binding mode.

## Figures and Tables

**Table 1:** ^1^H-NMR Chemical shifts (δ, ppm) for CH protons of β-CD alone, daidzein alone and their complexation induced shifts (CIS = **δ_complex_** − **δ_guest_**) in D_2_O at 30 °C.

β-CD	**CH Protons**	**H-1**	**H-2**	**H-3**	**H-4**	**H-5**	**H-6**
	δ alone	5.038	3.617	3.931	3.550	3.821	3.847
	CIS	−0.040	−0.052	−0.065	−0.026	−0.113	−0.097
Daidzein	CH protons	H-2	H-5	H-6	H-8	H-2′6′	H-3′5′
	δ alone	7.992	7.859	6.73	6.524	7.217	6.704
	CIS	−0.123	0.009	−0.022	−0.047	−0.049	−0.007

**Table 2:** ^1^H-NMR Chemical shifts (δ, ppm) for CH protons of β-CD alone, daidzin alone and their complexation induced shifts (CIS = **δ_complex_** − **δ_guest_**) in D_2_O at 30 °C.

β-CD	**CH Protons**	**H-1**	**H-2**	**H-3**	**H-4**	**H-5**	**H-6**
	δ alone	5.038	3.617	3.931	3.550	3.821	3.847
	CIS	−0.019	−0.021	−0.047	−0.012	−0.063	−0.050
Daidzin	CH protons	H-2	H-5	H-6	H-8	H-2′6′	H-3′5′
	δ alone	8.172	8.101	7.208	7.243	7.240	6.686
	CIS	−0.059	0.060	0.063	0.028	−0.019	0.031

**Table 3:** Diffusion coefficients (D) in D_2_O and association constants (K), log D_free_ (isoflavones alone 2 mM, diffusion/m^2^∙s^−1^), log D_+CD_ (isoflavones 2 mM with β-CD 2 mM), K (M^−1^).

Isoflavones	Daidzein	Daidzin	Puerarin
log D_free_	−9.27 ± 0.03	−9.34 ± 0.03	−9.35 ± 0.01
log D_+CD_	−9.37 ± 0.04	−9.38 ± 0.03	−9.39 ± 0.01
K	2843 ± 187	902 ± 66	781 ± 42
